# The Impact of Dance Interventions on Patients with Noninfectious Pulmonary Diseases: A Systematic Review

**DOI:** 10.3390/ijerph191711115

**Published:** 2022-09-05

**Authors:** Vikram Niranjan, Giampiero Tarantino, Jaspal Kumar, Diarmuid Stokes, Ray O’Connor, Andrew O’Regan

**Affiliations:** 1 UCD School of Public Health, Physiotherapy and Sports Science, University College Dublin, D04 V1W8 Dublin, Ireland; 2 NUS Centre for Cancer Research, Yong Loo Lin School of Medicine, National University of Singapore, Singapore 169857, Singapore; 3 UCD Library, University College Dublin, D04 V1W8 Dublin, Ireland; 4 School of Medicine, University of Limerick, V94 T9PX Limerick, Ireland

**Keywords:** dance, well-being, quality of life, pulmonary diseases, physical activity

## Abstract

Dance has been highlighted as one of the most enjoyable, safe, and feasible forms of physical activity, improving physical health, mental health, and general well-being, among various patients. Little is known about the effect and impact of dance interventions to improve health among patients with pulmonary diseases, and research lacks a robust synthesis of evidence. Therefore, this systematic review aimed to investigate the impact of dance intervention on patients with noninfectious pulmonary diseases. Following the PRISMA guidelines, six electronic databases were searched in May 2022. Of the 1308 unique records identified, seven studies (five quantitative, two qualitative) across four countries were included in this systematic review. Six studies investigated adult populations, and one study explored the effect of dance on children. The dance interventions lasted between 1 and 10 weeks. Overall, dance was perceived to have a broad range of physical/mental/social benefits, including quality of life, social cohesion, dyspnoea levels, balance, exercise tolerance, general well-being, and adherence to nutrition regimens. Furthermore, the dance session was the most enjoyable activity among children and adolescents with asthma. With available evidence, dance interventions are promising and may effectively improve health and well-being among patients with noninfectious pulmonary diseases. More organised and continuous long-term dance interventions in future may reveal a detailed impact on health outcomes.

## 1. Introduction

Chronic pulmonary diseases are one of the leading causes of morbidity and mortality globally [1], characterized by dyspnoea, exercise intolerance, decreased mobility, balance deficits, anxiety, depression, and poor quality of life, and are associated with worse clinical outcomes, such as hospital admission [2,3,4]. Physical activity (PA) is an effective pulmonary rehabilitation (PR) measure for improving health outcomes among patients with chronic pulmonary diseases. Recently, participation in alternative forms of physical activity as a type of exercise (i.e., soccer, walking, cardio exercise) has been shown to be beneficial for improving breathing efficiency and dyspnoea [5]. However, attendance and completion of PA programmes remains poor for people with chronic obstructive pulmonary disease (COPD) [6]. It is recommended to align PA programmes that are valued, enjoyed, and considered to be meaningful by the patient population. Not only are such programmes related to psychological flexibility, but also they may help to promote sustainable behavioural changes and encourage PA engagement among people living with COPD [7]. In this sense, dance has been acknowledged to be one such form that fulfils the criteria, leading to better quality of life. Dance has also been highlighted as one of the most enjoyable, safe, feasible, and engaging interventions in respiratory care [8].

Recent research evidence has shown that dance interventions can improve physical and mental health and general well-being among the elderly and patients with various medical conditions [9]. It has also shown that any form of dance activity may significantly improve strength, endurance, and balance levels among older adults. In this regard, three studies were conducted among patients with Parkinson’s disease, where different forms of dance, such as tango [10,11], American ballroom [11], ballet, contemporary, jazz, folk, and ballroom [12], were used and compared. Findings from these studies showed that any type of dance can be considered a feasible activity for adults with neurological conditions, which may improve their health outcomes. Furthermore, dance has been associated with a reduced risk of mortality related to cardiovascular conditions and diseases [13] and improved endothelial function as well as exercise capacity in patients with chronic heart failure [14]. Dance has also been reported to be helpful in improving gait coordination and health-related quality of life in patients with stroke [12,15]. Another systematic review, by Koch and colleagues, demonstrated that dance improves the quality of life among patients with chronic conditions [16]. It has been shown that dance is also a valuable form of physical activity to improve functional adaptations, balance, and gain control in older adults with and without chronic conditions (i.e., Parkinson’s disease) [10,16,17,18,19,20,21]. Alongside the physical benefits, recent experimental studies have shown that dance interventions may also be beneficial for mental health outcomes. It has been shown that dance reduces depression levels among adults and adolescents [17,22]. These findings were also corroborated by a recent systematic review that showed that dance movement therapy may reduce the psychological distress among vulnerable populations [16]. Overall, dance has been widely acknowledged to be equally and occasionally more effective than other types of PA for improving health outcomes [5].

However, although singing and dance have become increasingly popular forms of physical activity among people with chronic respiratory conditions and diseases [8], there is limited understanding of the extent to which dance interventions are effective and impactful, and research lacks a robust synthesis of evidence. Therefore, this study aimed to systematically review the literature to investigate the impact of dance interventions on patients with noninfectious pulmonary diseases as part of a feasibility study understanding the impact of dance on the health and well-being of patients with pulmonary fibrosis.

## 2. Materials and Methods

To address our research question, a systematic review was conducted to examine the available literature regarding the impact of dance intervention on patients with pulmonary diseases. Such a design would help us synthesise the current status of the literature and understand whether scientific findings are consistent.

### 2.1. Study Selection Criteria

Studies that met all of the following criteria were included in the review: (1) language: articles written in English; (2) dance-based interventions; (3) study subjects: patients with noninfectious pulmonary diseases of any age, design: experimental pre–post, quantitative, and qualitative cross-sectional or longitudinal studies; (4) study time frame: any article published until March 2022; (5) outcome: mental health (e.g., anxiety, depression, well-being, emotional, physical health, quality of health, quality of life scale, breathing, and efficiency; (6) article type: peer-reviewed publications; (7) time window of search: from the inception of an electronic bibliographic database to May 2022.

Studies were excluded from the review if they: (1) focused on any non-dance-based intervention (PA, yoga, etc.); (2) were not peer-reviewed (i.e., conference proceeding, letters to editors, etc.); and (3) investigated infectious pulmonary diseases.

### 2.2. Search Strategy and Sources of Evidence

Following the PRISMA guidelines, six different electronic databases (PubMed, CINAHL, SPORTDiscus, Web of Science, EMBASE, and PsycINFO) were searched in May 2022 to locate potentially relevant articles. First, we conducted a preliminary search study in PubMed and Medline searching article titles, abstracts, keywords, and subject headings to guide the development of our search strategy. Second, we included the identified keywords and subject headings from the search strategy across all databases being used. Finally, we looked at the reference lists from the articles selected for the review. The use of the MeSH terms and the thesaurus was also employed to broaden the search strategy. A faculty librarian also provided suggestions and verifications regarding the appropriate syntax and the adaptation of search strategies across databases. Search terms were developed for each databases using the PICO framework [23] and included three main concepts: (i) noninfectious pulmonary conditions, (ii) dance, and (iii) health and well-being. The final list of the keywords used is provided in Supplementary Materials. A pair of reviewers read each paper and met at multiple stages throughout the reviewing process to discuss any discrepancies and changes in eligibility criteria that emerged. Any existing discrepancies regarding which articles to include or exclude and/or why were deemed a “conflict” and subsequently sent to a third independent reviewer, who arbitrated discrepancies and made the final decision.

### 2.3. Data Extraction and Synthesis

A standardised data extraction form was used to collect the following methodological and outcome variables from each article: author, year, country, setting, study design, data collection method, sample size, age range, gender, health condition, intervention, outcome, and main findings. One author extracted the data from the studies (GT), which were confirmed and checked by the principal investigator (VN). To provide a more robust and comprehensive synthesis, the quantitative estimates reported in the studies were converted into effect sizes (Cohen’s *d*). If a study provided neither the raw data (mean and standard deviation) nor the effect size, a number of different metrics were used to calculate the effect size. Specifically, a few studies reported the median changes and the interquartile ranges. These values were converted and approximated into mean and standard deviations following the Hozo, Djulbegovic, and Hozo [24] formulas. The means and standard deviations were subsequently converted into effect size using Cohen’s formula (difference in means divided by the pooled standard deviation). The following scale of magnitude was used to assess the effect sizes: <0.19 = trivial; 0.20 to 0.49 = small; 0.50 to 0.79 = moderate; and ≥0.80 = large [25]. Given the small number of studies included in this systematic review that employed a qualitative design to collect data, a thematic analysis was deemed not applicable. Therefore, the qualitative findings reported in the studies were narratively synthesised alongside the quantitative results.

### 2.4. Study Quality Assessment

A tool developed by the National Heart, Lung, and Blood Institute was used to assess the quality of the studies [26]. This tool was developed to assess the quality of studies that employed a before–after design with no control group and observational cohort and cross-sectional studies. This assessment tool rates each study based on 12 criteria. For each criterion, a score of 1 was assigned if “yes” was the response, whereas a score of 0 was assigned otherwise (i.e., if an answer of “no”, for not applicable, “not reported”, or “cannot determine”. The quality was rated as follow: 9 to 12 = good quality; 5 to 8 = fair quality; 1 to 4 = poor quality. Two co-authors of this review independently conducted the study quality assessment, with discrepancies resolved through discussion with a third coauthor.

## 3. Results

**Figure 1:**
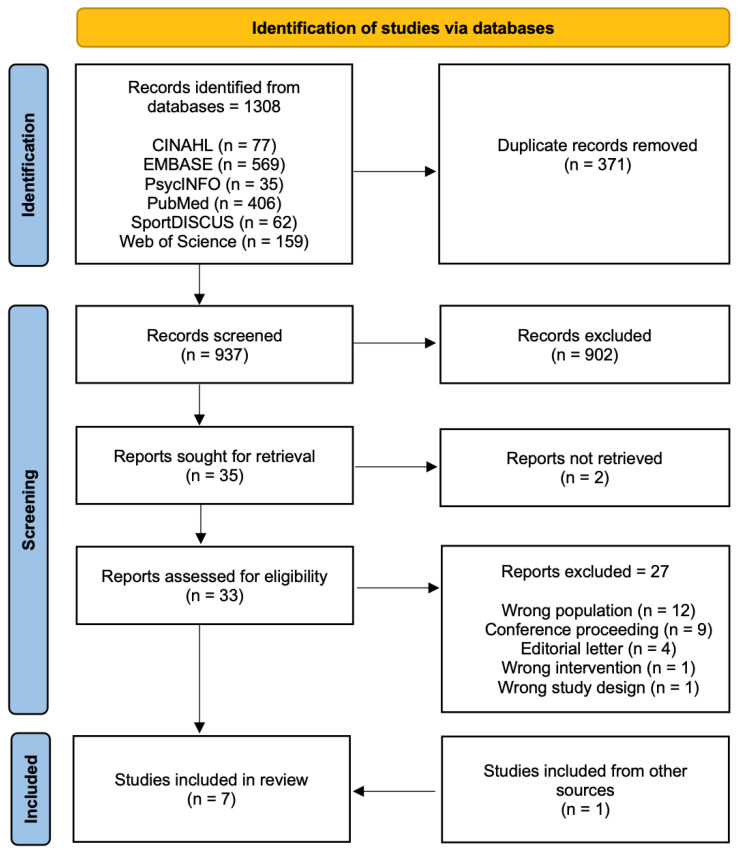
PRISMA chart.

The systematic search strategy identified 1308 potentially relevant articles, of which 371 were removed as duplicates. Of the remaining 937 articles, 902 did not meet the eligibility criteria and, therefore, were excluded from this review. Following screening and detailed assessment of the full text, a further 29 articles were excluded for different reasons (wrong population, conference proceeding, wrong intervention, and wrong design), resulting in six studies being deemed suitable for this systematic review. A manual search of the reference list of the articles included was performed, and one more article was deemed suitable for inclusion (Figure 1).

### 3.1. Overview of the Studies

As regards the seven studies included in this systematic review, all were published in the last 3 years [27,28,29,30,31,32], except one [33], which was published in 2005. Five studies investigated adults [27,28,29,30,31], whereas one study investigated children [32], and one final article investigated a population of people aged 17 years old or older [28]. All the seven studies were conducted in urban areas. Across the studies included in this systematic review, three were conducted in the UK [27,28,29], two in the US [32,33], and one in Uganda [31], and one in Canada [30]. Four studies employed a pre-/post-quantitative study design [28,30,32,33], one study employed a mixed-method approach with a pre-/post-quantitative study design and qualitative analysis post-intervention [29], and two studies gathered qualitative data after the intervention only [27,31]. In terms of sample size, six studies had a sample of fewer than or equal to 20, whereas one study had a sample size of 42 [33]. Finally, the types of pulmonary conditions differed across the studies’ participants. Among the 110 total participants who were involved in the studies, the majority of them (38.2%) had cystic fibrosis. Moreover, 27.3% of the participants had COPD, and 19.1% of the total sample had asthma. The remaining participants had post-tuberculosis lung diseases, post-infectious lung diseases, pulmonary fibrosis, and bronchiectasis. The type of condition was not reported in 7.3% of the participants (Table 1).

### 3.2. Overview of the Risk of Bias

The quality assessment of the study risk of bias is presented in Table 2.

Of the seven studies included in this review, six (86%) had samples that were considered representative of the study population, whereas in one study, this information could not be determined [29]. Seventy-one percent (*n* = 5) had an overall fair quality, whereas two studies had a good quality with minimal missing information.

### 3.3. Study Findings

As summarised in Table 3, the study findings investigated during the interventions can be divided in two main groups: (i) physical health and (ii) mental health.

#### 3.3.1. Physical Health findings

Five studies [28,29,30,31,32] investigated the benefit of dance activities on physical health outcomes, which included functional exercise capacity, respiratory capacity, and other physical health-related measures (e.g., heart rate monitoring, perceived health benefits, ratings of perceived exertion).

***Functional Exercise Capacity***. Three studies investigated the benefits of dance classes on functional exercise capacity [28,29,30], which is one of the key outcomes of pulmonary function among patients with lung diseases, and it is commonly measured by walking tests. The common tests used in the studies included in this systematic review involved: (i) Incremental Shuttle Walk Test (ISWT), (ii) 6-Minute Walk Test (6MWT), Time Up and Go Test (TUG), and 30-Second Sit-to-Stand Test (30-STS). Wshah et al. [30] found statistically significant small positive differences in relation to 6MWT (*d* = 0.26; *p* = 0.034), whereas Harrison et al. [29] reported moderate-sized positive differences (*d* = 0.58), but they did not provide the significance level. Finally, insufficient data to calculate the effect size were reported in one study [28]. Harrison et al. [29] found also large negative differences in the TUG Test (*d* = −1.09) and large positive differences in the 30-STS (*d* = 2.59). However, in both cases, the statistical significance levels were not reported. Gardiner et al. [28] found moderate-sized, although no-statistically significant, positive differences in the ISWT (*d* = 0.73, *p* = 0.14) and the handgrip (median change = +1 kilogramme, *p* = 0.29) after participating in the intervention. Regarding the balance, in Wshah et al. [30], large-sized statistically significant differences were found for the BEST-est (*d* = 1.29; *p* < 0.001) and in the ABC Scale (*d =* 0.51, *p =* 0.007). Moreover, small positive nonstatistically significant differences were found for the BBS (*d* = −0.12; *p* = 0.079). Although nonstatistically significant, negative trivial differences were found in the step count (*d* = −0.12; *p* = 0.19).

***Respiratory Capacity***. Two studies [28,29] investigated the COPD capacity using the COPD Assessment Tool. In both articles, nonstatistically significant small-sized negative differences were found (*d* = −45, *p* = 0.46; *d* = −0.32, *p* = not available). These two studies also investigated the differences in the dyspnoea levels after attending the dance intervention. Contrasting results were reported in the two studies, with one article [28] finding nonstatistically significant large-sized positive differences in *d* = 0.90 (*p* = 0.31), whereas statistically significant improvements in the dyspnoea levels, with positive moderate-sized differences, were found in the other study (*d* = 0.71, *p* = 0.001) [30].

***Other Physical Health Findings***. Wshah et al. [30] investigated the fatigue levels among the participants. Findings from this study revealed small-sized positive differences in the fatigue levels after participating in the dance intervention. However, these results were nonstatistically significant (*d* = 0.32, *p* = 0.077). One study by Schwartz et al. [32] compared five different activities (walking, resistance circuit, gamified dance, gamified obstacle course, and step test) among children with asthma and measured the in-task score regarding the ratings of perceived exertion (RPE) and heart rate (HR) reserve. Findings from this study revealed that dance reported the lowest score in the percentage of HR reserve and the second lowest score on RPE.

***Synthesis of Qualitative Evidence.*** One study [31] used semistructured interviews to investigate the potential benefits of music and dance in chronic respiratory diseases based in Uganda. Findings from this study revealed that the perceived physical and health improvements were primarily attributed to the dancing and singing activities. The main themes that emerged, in relation to the health outcomes, comprised improved breathing capacity and sputum coming out much easier. As this dancing activity was also associated with a singing activity, patients voiced that this combination of dance and singing “pushed and expanded the lungs” (p. 7, [31]) and made them “feel they breathed very well” (p. 5, [31]). In another exploratory qualitative cross-sectional study, Philip et al. (2020) [27] performed thematic analysis to investigate the perceptions of participants of a community dance group for people with chronic pulmonary diseases. The participants reported perceived physical health benefits, such as reduced disease severity, “until I was doing the exercise it was bad” (p. 7, [27]); improved breathing efficiency, “I think my breathing is better” (p. 1, [15]); and improved function, “mobility, I think, it’s one of the things that I have found so much better. Because when I joined, there were lots of things I found really difficult” (p. 1, [27]). Finally, in one study [29], informal group discussions were conducted to provide insights into the dance classes. Harrison et al. [29] found that participants in the dance programme contributed to promote “a holistic sense of well-being” (p. 5, [29]), with the participants reporting that during the dance activities, they forgot to be ill and concentrated on the dance movements, rather than their breathing issues. The participants in this study also reported physical benefits of the dance classes, stating that they “noticed definition on muscles on arms and legs” (p. 5, [29]).

#### 3.3.2. Mental Health Findings

All studies included in this systematic review investigated the mental health benefits of dance, which could be divided into: (i) anxiety levels, (ii) depression levels, and (iii) other mental health outcomes.

***Anxiety***. Two studies [28,30] investigated anxiety using the Hospital Anxiety Score (HADS Anxiety), whereas one study [29] used the Generalised Anxiety Disorder Assessment (GAD-7). Gardiner et al. [28] reported nonstatistically significant trivial positive differences (*d* = 0.07, *p* = 0.59), and Wshah et al. [30] reported small-sized, although nonstatistically significant, negative differences in the anxiety score after attending the dance classes (*d* = −0.40, *p* = 0.072). Finally, using the GAD-7 questionnaire, Harrison et al. [29] found moderate-sized negative differences (*d* = −0.77) regarding the anxiety scores. However, it was impossible to determine the significance level as the *p*-value was not reported.

***Depression***. Three studies investigated changes in depression levels after the dance classes [28,29,30]. Two studies used the HADS Depression questionnaire and found nonstatistically significant differences. In particular, Gardiner et al. [28] found trivial-sized negative differences (*d* = −0.03, *p* = 0.71), whereas Wshah et al. [30] found small-sized negative differences on the depression symptoms (*d* = −0.27, *p* = 0.251). Finally, although the significance level was not reported, Harrison et al. [29] found large-sized negative differences in the depression symptoms (*d* = −1.84).

***Other Mental Health Findings***. Six studies investigated other mental health and social benefits of the dance intervention. Goodill [33] employed the Profile of Mood States questionnaire (POMS) to measure the mood state in participants with cystic fibrosis. However, no differences were found after participation in three small group dance sessions (not enough data were reported to calculate the effect size and the *p*-value). Harrison et al. [29] used the Multidimensional Assessment of Interoceptive Awareness to investigate information about the body awareness. This questionnaire comprised eight subscales (noticing, not distracting, not worrying, attention regulation, emotional awareness, self-regulation, body listening, and trusting). Although the statistical significance levels were not reported, the effect sizes ranged from *d* = −2.09 for noticing to *d* = 1.98 for trusting, with an average small effect size of *d* = 0.21, suggesting that the body awareness increased after participation in the dance intervention. Wshah et al. [30] found statistically significant large-sized positive differences (*d* = 0.85, *p* = 0.001) in the health-related quality of life and moderate-sized positive differences in the emotional function (*d* = 0.60, *p* = 0.001). In their comparative investigation, Schwartz et al. [32] found that dance was perceived by the participants as the most enjoyable activity.

***Synthesis of Qualitative Evidence.*** In two qualitative studies, refs. [27,31] semistructured interviews were used to investigate the social benefits, enjoyment, and mental health of participants after the dance intervention. In a study by Philip et al. (2021) [31], participants reported that not only music and dance were central components of their daily life, but also they made participants “feeling happier, and more connected with the world” (p. 4, [31]). Participants voiced the social aspect that they felt during the intervention, describing the dance and singing sessions as an enjoyable opportunity to “join other people” and “support each other” (p. 5, [31]).

Meanwhile, in Philip et al. (2022) [27], thematic analysis revealed that dance was perceived as an enjoyable activity and deemed to be an important part of participants’ lives. Moreover, the participants voiced that they attended the intervention for the “social side as well, because it was very nice to meet people with the same basic difficulties” (p. 5, [27]). The social aspect of this intervention was also perceived as an important outcome that benefited their mental health, as the participants felt that they were “supporting each other” (p. 5, [27]) to create a supportive atmosphere within the group.

Finally, Harrison et al. [29], in their mixed-methods investigation, found that dance had a positive impact on participants’ social life, as they had the opportunity to know everybody and share the classes with people experiencing the same problems. Moreover, the participants reported that the dance classes were very “enjoyable” (p. 5, [29]) and contributed to make them feel relaxed, like “a kid in the playground” (p. 5, [29]).

## 4. Discussion

The purpose of this systematic review was to investigate the available literature regarding the benefits of dance intervention on patients with noninfectious pulmonary diseases. According to the majority of the studies that employed quantitative designs to investigate the impact of dance on patients with pulmonary conditions, dance activities had potentially positive impacts on participants’ physical and mental health. In terms of physical health outcomes, our findings revealed that dance may have potential improvements in functional exercise and respiratory capacity and may reduce fatigue and breathlessness levels. Among mental health outcomes, findings from this systematic review showed that dance may reduce anxiety and depression levels and improve health-related quality of life and emotional function. However, the small sample sizes might have affected the significance levels. The quantitative findings are further supported by the two qualitative studies included in this review, which highlighted the extent to which participants deemed the dance activities to be helpful for their physical and mental health, as well as their socialisation needs. Previous research has shown that not only is dance a feasible and low-cost activity for people living with chronic lung diseases [8], but also its benefits are well perceived among participants [20].

To our knowledge, this is the first study that systematically reviewed the existing literature regarding the impact of dance activities on people with chronic pulmonary conditions. Overall, the findings from this systematic review highlighted the limited evidence available in this research area. The number of articles included in this review and the year in which they were published are themselves important findings, which suggest that this research area is still novel and needs further exploration.

However, considering that people living with chronic pulmonary conditions fail to engage in regular PA due to a number of reasons (hospitalisations, loneliness, access to PA facilities) [34], our findings can be framed within the context of two major concepts: (i) PA level for such group of people and (ii) policy implementation for promoting PA, including dance, to people who cannot access sports facilities, with particular emphasis on those whose PA levels have been affected by the recent pandemic. In this sense, our results revealed that dance, as a form of physical activity, can be an excellent and enjoyable type of exercise for people living with chronic lung conditions. Gardiner et al. [28] showed that the completion rate was 34.5% higher in a community-based PR programme that included Latin-dance-based exercise as compared with the conventional programme (PA: walking, step-ups, sit-to-stand, and cycling and strengthening exercises using weights) for people with COPD. It is our belief that other groups of people might benefit from this type of PA. In this sense, the findings from our study align well with those reported by recent systematic reviews investigating the impact of dance intervention on other forms of clinical conditions. Their results revealed that dance may have beneficial effects on physical and mental health among children with disabilities [35] and people living with Parkinson’s disease [20], breast cancer, stable heart failure, and diabetes [36]. Our findings, therefore, corroborate previous research evidence, suggesting that dance can be a helpful activity that may have a positive impact on the health and well-being of people with chronic pulmonary conditions [27,28,29,30]. Furthermore, when considering the extent to which this population is at high risk during the current pandemic, results from this systematic review suggest that home-based dance activities may help to reduce the negative impact of isolation due to the closure of sports facilities, which has been acknowledged to be one of the major barriers to PA participation in individual and group PA. Finally, when considering the degree to which research evidence has shown how low levels of PA exacerbate the negative impact on mental health among people, dance activities become an excellent form of PA that can be delivered remotely.

In terms of policy implications, our findings revealed that not only are dance interventions feasible, but also they are reproducible to larger scales. Wshah et al.’s [30] findings showed that the dance intervention was attended by 78% of the initial people contacted, the majority of whom indicated that they were motivated to continue with the programme if it had been carried on by the research group, as their mood and enjoyment had significantly improved. Moreover, despite dealing with the current COVID-19 pandemic, Schwartz et al. [32] successfully delivered in a safe clinical environment, with a low dropout rate, the comparative intervention, including dancing activities, to children living with asthma. In this sense, the findings from this systematic review support the view that it is necessary to formulate public health policies related to PA promotion, in particular during the current pandemic, to improve the mental, physical, and social health of vulnerable people. As research has also shown the mental and physical benefits of home-based PA among healthy older adults [37,38], cancer survivors [39], and people with Parkinson’s disease [40], it is essential that policymakers, researchers, and practitioners embrace such a piece of evidence and support and encourage PA programmes aimed at developing: (i) home-based PA and (ii) outdoor PA conducted in natural and safe environments. Such a policy implementation will enable vulnerable groups of people (such as people living with chronic pulmonary disease, heart failure, Parkinson’s disease, and older adults etc.) to meet the daily levels of PA recommended by the World Health Organisation [41]. For example, within the Irish context, national guidelines emphasise the promotion of PA among older people, children with disabilities [42], and people living with chronic conditions, who are considered vulnerable. In this regard, Sport Ireland has developed policies aiming to promote sport and PA among these populations and encouraged policymakers to build capacity and create new opportunities [43]. Here, dance interventions could be a suitable form of home-based PA, setting an example of collaboration among different policymakers and stakeholders. Furthermore, by planning and designing innovative, safer, and favourable PA measures that can be actively implemented in any context, unnecessary negative effects on mental and physical health may be avoided.

An interesting, yet not surprising, finding from this systematic review is the discrepancy in the methodologies used to investigate the physical and mental health of people living with chronic pulmonary diseases. Using common measurement methods (questionnaires and tools) would have enabled us to employ a meta-analytic approach to synthesise quantitative evidence. This is not a limitation strictly related to our study. Rather, it is a limitation that embraces research conducted in this specific area. Although validated instruments exist to assess mental and physical health in people with chronic pulmonary conditions (i.e., 6MWT, CAT, HADS questionnaire, etc.), their use is limited to the research questions being posed. Additionally, as qualitative studies have revealed a deeper understanding of the holistic health benefits of dance interventions, developing tools to measure such impact is necessary, and that would enable performing an in-depth qualitative synthesis.

Finally, the physical and mental health benefits of dance activities have been widely reported among other cohorts of population [9,44,45,46], showing the great interest that research has demonstrated toward this form of PA. However, in relation to people living with chronic pulmonary conditions, research is still limited. Our findings reported contradictory results from the studies included in this systematic review, which suggests that future research ought to focus on corroborating evidence reported in this study by implementing and conducting new research studies on dance activities for patients with noninfectious pulmonary diseases, preferably using the same instrument to measure mental and physical health outcomes.

## 5. Conclusions

This review explored the current status of the literature regarding the impact of dance activities on the mental and physical health of people with chronic lung conditions. Regarding physical health, this study found that dance classes had positive and significant benefits on walking distance, balance, and dyspnoea levels. In relation to mental health, our findings revealed no changes in anxiety and depression levels after attending the dance classes, which might be due to the small sample sizes. However, significant changes were found in relation to the quality of life and emotional function. Furthermore, our findings showed that dance was an enjoyable activity for the participants to socialise and overturn the negative effects of physical inactivity. Results from this review reveal the limited evidence available in this area and suggest that further investigations are needed to corroborate previously reported findings and provide more robust evidence regarding the benefits of dance on people with chronic lung conditions.

## Figures and Tables

**Table 1:** Summary of studies included in this systematic review.

Author(s), Year	Country	Setting	Study Design and Data Collection Method	Sample Characteristics			Health Condition	Intervention		
				Size	Age	Gender		Type	Weekly Sessions	Total Duration
Gardiner et al., 2020 [28]	UK	Urban health Centre	Experimental pre–post. Survey	4	69 ± N/A	4F	4 × COPD	Latin-style dance	2 × 60 min	8 weeks
Goodill 2005 [33]	USA	Urban hospital	Experimental pre–post. Survey	42	17 or older	N/A	CF	Dance and movement therapy	1 × 60 min	3 sessions
Harrison et al., 2020 [29]	UK	Urban health centre	Experimental mixed method. Survey	8	70 ± 24	7F, 1M	N/A	Dance	1 × 90 min	10 weeks
Philip et al., 2021 [31]	Uganda	Urban health centre	Cross-sectional (qualitative). Semistructured interviews	11	43 (20–63)	8F, 3M	6 × PTBLD 1 × post-infection LD 2 × asthma 1 × COPD 1 × pulmonary fibrosis	Singing and dancing	1 × 20–40 min	N/A
Philip et al., 2020 [27]	UK	Urban health centre	Cross-sectional (qualitative). Semi-structured interviews	8	75 ± N/A	N/A	5 × COPD 2 × asthma 1 × bronchiectasis	Group dance	1 × 75 min	N/A
Schwartz et al., 2022 [32]	USA	Urban	Experimental pre–post. Survey	17	11.1 ± 0.6	8F, 9M	Asthma	Just dance	1 × 5 min	1 session
Wshah et al., 2019 [30]	Canada	Urban health centre	Experimental pre–post. Survey	20	73.4 ± 7.6	14F, 6M	20 × COPD	Dance	2 × 60 min	8 weeks

Notes: PTBLD = post-tuberculosis lung disease; LD = lung disease; COPD = chronic obstructive pulmonary disease; CF = cystic fibrosis; N/A= not available.

**Table 2:** Quality assessment of included studies in this systematic review.

Quality Assessment Tool for before–after (Pre–Post) Studies with No Control Group							
Criteria	Gardiner et al., 2020 [28]	Goodill 2005 [33]	Harrison et al., 2020 [29]	Philip et al., 2020 [31]	Philip et al., 2021 [27]	Schwartz et al., 2022 [32]	Wshah et al., 2019 [30]
1. Study question clear	Yes	No	Yes	Yes	Yes	Yes	Yes
2. Selection of the population clearly described	Yes	CD	No	Yes	Yes	Yes	Yes
3. Participant representative of the population	Yes	Yes	CD	Yes	Yes	Yes	Yes
4. Participant meeting entry criteria	Yes	Yes	CD	Yes	Yes	Yes	Yes
5. Adequate sample size	No	Yes	No	Yes	Yes	Yes	Yes
6. Intervention clearly described and delivered	Yes	Yes	Yes	Yes	Yes	Yes	Yes
7. Outcome measures clearly defined and valid	Yes	Yes	Yes	NA	NA	Yes	Yes
8. People assessing the outcome blinded to exposure	No	No	CD	No	No	No	No
9. Loss to follow-up less than 20%	Yes	No	Yes	Yes	Yes	Yes	Yes
10. Adequate analysis	Yes	Yes	Yes	Yes	Yes	Yes	Yes
11. Outcome measures taken multiple times before and after intervention	No	Yes	No	No	No	NA	NA
12. Intervention taken at a group level with appropriate statistical analysis	NA	No	Yes	Na	NA	Yes	NA
**Total Score**	**8**	**7**	**6**	**8**	**8**	**10**	**9**
**Quality Rating**	**Fair**	**Fair**	**Fair**	**Fair**	**Fair**	**Good**	**Good**

Notes: CD, cannot determine; NA, not applicable. A sample size was deemed to be adequate if larger than 10 participants. Reference: National Heart, Lung, and Blood Institute website. Quality Assessment Tool for Before–After (Pre–Post) Studies with no Control Group. Available online at: https://www.nhlbi.nih.gov/health-topics/study-quality-assessment-tools (accessed on 9 June 2022).

**Table 3:** Study findings from included studies in this systematic review.

Study	Physical Health Findings			Mental Health Findings		
	Functional Exercise Capacity	Respiratory Capacity	Other Physical Health Findings	Anxiety	Depression	Other Mental Health Findings
Gardiner et al., 2020 [28]	ISWT, 6MWT, handgrip	CAT, dyspnoea		HADS Anxiety	HADS Depression	
Goodill 2005 [33]						POMS
Harrison et al., 2020 [29]	6MWT, TUG, 30-STS	CAT	Physical benefits	GAD-7	PHQ-9	MAIA, social benefits, enjoyment
Philip et al., 2021 [31]			Physical health, health benefits			Social benefits, appropriateness, enjoyment, mental health
Philip et al., 2020 [27]						Experience of respiratory illness and impact on life; experience of dance group; perceived impacts of dance group participation
Schwartz et al., 2022 [32]			In-task RPE, HR reserve			In-task mood and enjoyment
Wshah et al., 2019 [30]	6MWT, BEST-est, BBS, ABC Scale, step count	Dyspnoea	Fatigue	HADS Anxiety	HADS Depression	HR-CRDQ, emotional function

Notes: COPD = chronic obstructive pulmonary disease; CF = cystic fibrosis; ISWT = Incremental Shuttle Walk Test; 6MWT = 6-Minute Walk Test; CAT = COPD Assessment Tool; Handgrip = handgrip measured in kg; HADS Anxiety = Hospital Anxiety Score; HADS Depression = Hospital Depression Score; BEST-est = Balance Evaluation System Test; BBS = Berg Balance Scale; ABC = Activities-Specific Balance Confidence Scale; HR-CRDQ = Health-Related Quality of Life Using Chronic Respiratory Disease Questionnaire; TUG = Timed Up and Go Test; 30-STS = 30-Second Sit-to-Stand Test; PHQ-9 = Patient Health Questionnaire-9; MAIA = Multidimensional Assessment of Interoceptive Awareness; HR = Heart Rate Monitoring; GAD-7 = Generalised Anxiety Disorder Assessment-7; POMS = Profile of Mood States.

## Supplementary Materials

The following supporting information can be downloaded at: https://www.mdpi.com/article/10.3390/ijerph191711115/s1.
