# Supplementary material for: The Impact of Dance Interventions on Patients with Noninfectious Pulmonary Diseases: A Systematic Review
Source: Int J Environ Res Public Health. 2022 Sep 5;19(17):11115. doi: 10.3390/ijerph191711115 (PMC9518039; doi:10.3390/ijerph191711115)
Supplement: Supplementary file 1 [file ijerph-19-11115-s001.zip › ijerph-1857858-supplementary.pdf]

PUBMED

COPD OR "Chronic obstructive pulmonary disease" OR "lung cancer" OR “Lung Neoplasm*” OR “lung tumor*”OR Asthma OR “Chronic bronchitis” OR Emphysema OR “cystic fibrosis” OR ILF OR “idiopathic lung fibrosis” OR “Idiopathic pulmonary fibrosis” OR “fibrosing alveolitis” OR “pulmonary fibrosis” OR “lung fibrosis” OR "Lung injuries" OR "Silicosis” OR “Sleep Apnea” OR “Sleep Apnoea” OR “Pulmonary hypertension” OR Sarcoidosis OR “Pulmonary embolism” OR “lung embolism” OR “Pulmonary arterial hypertension” OR PAH OR “Primary ciliary dyskinesia” OR PCD OR Pneumothorax OR “occupational lung diseases” OR Lymphangioleiomyomatosis OR “Interstitial lung disease” OR “Hypersensitivity pneumonitis” OR “Eosinophilic granulomatosis with polyangiitis” OR Mesothelioma OR “Vaping Use-Associated Lung Injury” OR “E-cigarette Use-Associated Lung Injury” OR EVALI OR “Vaping associated pulmonary illness” OR VAPI OR “Cryptogenic organizing pneumonia” OR COP OR “Black lung disease” OR Anthracosis OR “Bronchopulmonary dysplasia” OR “Lung Dysplasia” OR BPD OR “Bronchiolitis obliterans” OR Bronchiolitis OR “Chronic obstructive airways disease” OR Asbestosis OR “Lung injury” OR “Coal workers' pneumoconiosis” OR CWP OR “Primary ciliary dyskinaesia” OR COAD OR Pneumonia OR “Lung Disorder*” OR "Lung Disease*" OR "Pulmonary Disease, Chronic Obstructive"[Mesh] OR "Lung Neoplasms"[Mesh] OR "Asthma"[Mesh] OR "Bronchitis, Chronic"[Mesh] OR "Pulmonary Emphysema"[Mesh] OR "Cystic Fibrosis"[Mesh] OR "Pulmonary Fibrosis"[Mesh] OR "Idiopathic Pulmonary Fibrosis"[Mesh] OR "Silicosis"[Mesh] OR "Sleep Apnea Syndromes"[Mesh] OR "Hypertension, Pulmonary"[Mesh] OR "Sarcoidosis, Pulmonary"[Mesh] OR "Pulmonary Embolism"[Mesh] OR "Pulmonary Arterial Hypertension"[Mesh] OR "Pneumothorax"[Mesh] OR "Lymphangioleiomyomatosis"[Mesh] OR "Lung Diseases, Interstitial"[Mesh] OR "Mesothelioma"[Mesh] OR "Cryptogenic Organizing Pneumonia"[Mesh] OR "Anthracosis"[Mesh] OR "Bronchopulmonary Dysplasia"[Mesh] OR "Bronchiolitis Obliterans"[Mesh] OR "Bronchiolitis"[Mesh] OR "Asbestosis"[Mesh] OR "Lung Injury"[Mesh] OR "Lung Diseases"[Mesh] OR "Lung Injury"[Mesh] OR "Pneumonia"[Mesh]

AND

Danc* OR "Dancing"[Mesh]

AND

Wellbeing OR well-being OR “mental health” OR “physical health” OR health OR “quality of life” OR “QoF scale” OR “Breathing efficiency” OR Balance OR Gait OR anxiety OR fitness OR depression OR “exercise capacity” OR “muscle tone” OR “shortness of breath” OR Dyspnea OR dyspnoea OR emotion* OR tiredness OR Fatigue OR Energy OR Worry OR satisfaction OR happiness OR vitality OR "Mental Health"[Mesh] OR "Health"[Mesh] OR "Quality of Life"[Mesh] OR "Gait"[Mesh] OR "Anxiety"[Mesh] OR "Depression"[Mesh] OR "Muscle Tonus"[Mesh] OR "Dyspnea"[Mesh] OR "Emotions"[Mesh] OR "Fatigue"[Mesh] OR "Personal Satisfaction"[Mesh] OR "Happiness"[Mesh]

CINAHL

COPD OR "Chronic obstructive pulmonary disease" OR "lung cancer" OR “Lung Neoplasm*” OR “lung tumor*”OR Asthma OR “Chronic bronchitis” OR Emphysema OR “cystic fibrosis” OR ILF OR “idiopathic lung fibrosis” OR “Idiopathic pulmonary fibrosis” OR “fibrosing alveolitis” OR “pulmonary fibrosis” OR “lung fibrosis” OR "Lung injuries" OR "Silicosis" OR “Sleep Apnea” OR “Sleep Apnoea” OR “Pulmonary hypertension” OR Sarcoidosis OR “Pulmonary embolism” OR “lung embolism” OR “Pulmonary arterial hypertension” OR PAH OR “Primary ciliary dyskinesia” OR PCD OR Pneumothorax OR “occupational lung diseases” OR Lymphangioleiomyomatosis OR “Interstitial lung disease” OR “Hypersensitivity pneumonitis” OR “Eosinophilic granulomatosis with polyangiitis” OR Mesothelioma OR “Vaping Use-Associated Lung Injury” OR “E-cigarette Use-Associated Lung Injury” OR EVALI OR “Vaping associated pulmonary illness” OR VAPI OR “Cryptogenic organizing pneumonia” OR COP OR “Black lung disease” OR Anthracosis OR “Bronchopulmonary dysplasia” OR “Lung Dysplasia” OR BPD OR “Bronchiolitis obliterans” OR Bronchiolitis OR “Chronic obstructive airways disease” OR Asbestosis OR “Lung injury” OR “Coal workers' pneumoconiosis” OR CWP OR “Primary ciliary dyskinaesia” OR COAD OR Pneumonia OR “Lung Disorder*” OR "Lung Disease*" OR MH "Pulmonary Disease, Chronic Obstructive+" OR MH "Asthma+" OR MM "Bronchitis, Chronic" OR MH "Emphysema+" OR MM "Cystic Fibrosis" OR MM "Idiopathic Pulmonary Fibrosis" OR MH "Hypertension, Pulmonary+" OR MM "Sarcoidosis" OR MM "Pulmonary Embolism" OR MM "Pulmonary Arterial Hypertension" OR MM "Pneumothorax" OR MH "Lung Diseases, Interstitial+" OR MH "Mesothelioma+" OR MH "Cryptogenic Organizing Pneumonia" OR MM "Bronchopulmonary Dysplasia" OR MH "Bronchiolitis Obliterans+" OR MH "Bronchiolitis+" OR MH "Lung Injury+" OR MM "Anthracosis" OR MH "Lung Diseases+" OR MH "Lung Neoplasms+" MH "Pulmonary Fibrosis+" OR MH "Lung Injury+" OR MH "Pneumonia+"

AND

Danc* OR MH "Dancing+"

AND

Wellbeing OR well-being OR “mental health” OR “physical health” OR health OR “quality of life” OR “QoF scale” OR “Breathing efficiency” OR Balance OR Gait OR anxiety OR fitness OR depression OR “exercise capacity” OR “muscle tone” OR “shortness of breath” OR Dyspnea OR dyspnoea OR emotion* OR tiredness OR Fatigue OR Energy OR Worry OR satisfaction OR happiness OR vitality OR MH "Mental Health+" OR MH "Health+" OR MH "Quality of Life+" OR MH "Gait+" OR MH "Anxiety+" OR MH "Depression+" OR MH "Emotions+" OR MH "Fatigue+" OR MM "Worry" OR MM "Happiness" OR MH "Dyspnea+"

SPORTDIscus

COPD OR "Chronic obstructive pulmonary disease" OR "lung cancer" OR “Lung Neoplasm*” OR “lung tumor*”OR Asthma OR “Chronic bronchitis” OR Emphysema OR “cystic fibrosis” OR ILF OR “idiopathic lung fibrosis” OR “Idiopathic pulmonary fibrosis” OR “fibrosing alveolitis” OR “pulmonary fibrosis” OR “lung fibrosis” OR "Lung injuries” OR "Silicosis” OR “Sleep Apnea” OR “Sleep Apnoea” OR “Pulmonary hypertension” OR Sarcoidosis OR “Pulmonary embolism” OR “lung embolism” OR “Pulmonary arterial hypertension” OR PAH OR “Primary ciliary dyskinesia” OR PCD OR Pneumothorax OR “occupational lung diseases” OR Lymphangioleiomyomatosis OR “Interstitial lung disease” OR “Hypersensitivity pneumonitis” OR “Eosinophilic granulomatosis with polyangiitis” OR Mesothelioma OR “Vaping Use-Associated Lung Injury” OR “E-cigarette Use-Associated Lung Injury” OR EVALI OR “Vaping associated pulmonary illness” OR VAPI OR “Cryptogenic organizing pneumonia” OR COP OR “Black lung disease” OR Anthracosis OR “Bronchopulmonary dysplasia” OR “Lung Dysplasia” OR BPD OR “Bronchiolitis obliterans” OR Bronchiolitis OR “Chronic obstructive airways disease” OR Asbestosis OR “Lung injury” OR “Coal workers' pneumoconiosis” OR CWP OR “Primary ciliary dyskinaesia” OR COAD OR Pneumonia OR “Lung Disorder*” OR "Lung Disease*" OR DE "CHRONIC obstructive pulmonary disease" OR DE "LUNG cancer" OR DE "PULMONARY emphysema" OR DE "CYSTIC fibrosis OR DE "PULMONARY hypertension" OR DE "PULMONARY embolism" OR DE "PNEUMOTHORAX" OR DE "PULMONARY hypertension" OR DE "LUNG diseases" OR DE "PULMONARY fibrosis" OR DE "LUNG injuries" OR DE "PNEUMONIA"

AND

Danc* OR DE "DANCE"

AND

Wellbeing OR well-being OR “mental health” OR “physical health” OR health OR “quality of life” OR “QoF scale” OR “Breathing efficiency” OR Balance OR Gait OR anxiety OR fitness OR depression OR “exercise capacity” OR “muscle tone” OR “shortness of breath” OR Dyspnea OR dyspnoea OR emotion* OR tiredness OR Fatigue OR Energy OR Worry OR satisfaction OR happiness OR vitality OR DE "WELL-being" OR DE "MENTAL health" OR DE "HEALTH" OR DE "QUALITY of life" OR DE "BREATHING exercises" OR DE "GAIT disorders" OR DE "ANXIETY" OR DE "MENTAL depression" OR DE "MUSCLE tone" OR DE "DYSPNEA" OR DE "EMOTIONS" OR DE "FATIGUE" OR DE "VITALITY"

Web Of Science

(((ALL=(COPD)) OR ALL=("Chronic obstructive pulmonary disease")) OR ALL=("lung cancer")) OR ALL=(“Lung Neoplasm*”)) OR ALL=(“lung tumor*”)) OR ALL=(Asthma)) OR ALL=(“Chronic bronchitis”)) OR ALL=(Emphysema)) OR ALL=(“cystic fibrosis”)) OR ALL=(ILF)) OR ALL=(“idiopathic lung fibrosis”)) OR ALL=(“Idiopathic pulmonary fibrosis”)) OR ALL=(“fibrosing alveolitis”)) OR ALL=(“pulmonary fibrosis”)) OR ALL=(“lung fibrosis”)) OR ALL=("Lung injuries”)) OR ALL=(Silicosis)) OR ALL=(“Sleep Apnea”)) OR ALL=(“Sleep Apnoea”)) OR ALL=(“Pulmonary hypertension”)) OR ALL=(Sarcoidosis)) OR ALL=(“Pulmonary embolism”)) OR ALL=(“lung embolism”)) OR ALL=(“Pulmonary arterial hypertension”)) OR ALL=(PAH)) OR ALL=(“Primary ciliary dyskinesia”)) OR ALL=(PCD)) AND ALL=(Pneumothorax)) OR ALL=(“occupational lung diseases”)) OR ALL=(Lymphangioleiomyomatosis)) OR ALL=(“Interstitial lung disease”)) OR ALL=(“Hypersensitivity pneumonitis”)) OR ALL=(“Eosinophilic granulomatosis with polyangiitis”)) OR ALL=(Mesothelioma)) OR ALL=(“Vaping Use-Associated Lung Injury”)) OR ALL=(“E-cigarette Use-Associated Lung Injury”)) OR ALL=(EVALI)) OR ALL=(“Vaping associated pulmonary illness”)) OR ALL=(VAPI)) OR ALL=(“Cryptogenic organizing pneumonia”)) OR ALL=(COP)) OR ALL=(“Black lung disease”)) OR ALL=(Anthracosis)) OR ALL=(“Bronchopulmonary dysplasia”)) OR ALL=(“Lung Dysplasia”)) OR ALL=(BPD)) OR ALL=(“Bronchiolitis obliterans”)) OR ALL=(Bronchiolitis)) OR ALL=(“Chronic obstructive airways disease”)) OR ALL=(Asbestosis)) OR ALL=(“Lung injury”)) OR ALL=(“Coal workers' pneumoconiosis”)) OR ALL=(CWP)) OR ALL=(“Primary ciliary dyskinaesia”)) OR ALL=(COAD)) OR ALL=(Pneumonia)) OR ALL=(“Lung Disorder*”)) OR ALL=("Lung Disease*")

AND

ALL=(Danc*)

AND

(((((((((((((((((((((((((ALL=(Wellbeing)) OR ALL=(well-being)) OR ALL=(“mental health”)) OR ALL=(“physical health”)) OR ALL=(health)) OR ALL=(“quality of life”)) OR ALL=(“QoF scale”)) OR ALL=(“Breathing efficiency”)) OR ALL=(Balance)) OR ALL=(Gait)) OR ALL=(anxiety)) OR ALL=(fitness)) OR ALL=(depression)) OR ALL=(“exercise capacity”)) OR ALL=(“muscle tone”)) OR ALL=(“shortness of breath”)) OR ALL=(Dyspnea)) OR ALL=(dyspnoea)) OR ALL=(emotion*)) OR ALL=(tiredness)) OR ALL=(Fatigue)) OR ALL=(Energy)) OR ALL=(Worry)) OR ALL=(satisfaction)) OR ALL=(happiness)) OR ALL=(vitality)

EMBASE

COPD OR "Chronic obstructive pulmonary disease" OR "lung cancer" OR “Lung Neoplasm*” OR “lung tumor*” OR Asthma OR “Chronic bronchitis” OR Emphysema OR “cystic fibrosis” OR ILF OR “idiopathic lung fibrosis” OR “Idiopathic pulmonary fibrosis” OR “fibrosing alveolitis” OR “pulmonary fibrosis” OR “lung fibrosis” OR "Lung injuries” OR "Silicosis” OR “Sleep Apnea” OR “Sleep Apnoea” OR “Pulmonary hypertension” OR Sarcoidosis OR “Pulmonary embolism” OR “lung embolism” OR “Pulmonary arterial hypertension” OR PAH OR “Primary ciliary dyskinesia” OR PCD OR Pneumothorax OR “occupational lung diseases” OR Lymphangioleiomyomatosis OR “Interstitial lung disease” OR “Hypersensitivity pneumonitis” OR “Eosinophilic granulomatosis with polyangiitis” OR Mesothelioma OR “Vaping Use-Associated Lung Injury” OR “E-cigarette Use-Associated Lung Injury” OR EVALI OR “Vaping associated pulmonary illness” OR VAPI OR “Cryptogenic organizing pneumonia” OR COP OR “Black lung disease” OR Anthracosis OR “Bronchopulmonary dysplasia” OR “Lung Dysplasia” OR BPD OR “Bronchiolitis obliterans” OR Bronchiolitis OR “Chronic obstructive airways disease” OR Asbestosis OR “Lung injury” OR “Coal workers pneumoconiosis” OR CWP OR “Primary ciliary dyskinaesia” OR COAD OR Pneumonia OR “Lung Disorder*” OR "Lung Disease*" OR 'chronic obstructive lung disease'/exp OR 'lung cancer'/exp OR 'asthma'/exp OR 'chronic bronchitis'/exp OR 'emphysema'/exp OR 'lung tumor'/exp OR 'fibrosing alveolitis'/exp OR 'lung fibrosis'/exp OR 'silicosis'/exp OR 'pulmonary hypertension'/exp OR 'sarcoidosis'/exp OR 'lung embolism'/exp OR 'pulmonary hypertension'/exp OR 'ciliary dyskinesia'/exp OR 'pneumothorax'/exp OR 'lymphangioleiomyomatosis'/exp OR 'interstitial lung disease'/exp OR 'mesothelioma'/exp OR 'vaping associated lung injury'/exp OR 'anthracosis'/exp OR 'lung dysplasia'/exp OR bronchiolitis obliterans'/exp OR 'bronchiolitis'/exp OR 'asbestosis'/exp OR 'lung injury'/exp OR 'lung disease'/exp OR 'lung injury'/exp OR 'pneumonia'/exp

AND

Danc* OR 'dancing'/exp

AND

Wellbeing OR well-being OR “mental health” OR “physical health” OR health OR “quality of life” OR “QoF scale” OR “Breathing efficiency” OR Balance OR Gait OR anxiety OR fitness OR depression OR “exercise capacity” OR “muscle tone” OR “shortness of breath” OR Dyspnea OR dyspnoea OR emotion* OR tiredness OR Fatigue OR Energy OR Worry OR satisfaction OR happiness OR vitality OR 'wellbeing'/exp OR 'mental health'/exp OR 'health'/exp OR 'quality of life'/exp OR 'gait'/exp OR 'anxiety'/exp OR 'fitness'/exp OR 'depression'/exp OR 'muscle tone'/exp OR 'dyspnea'/exp OR 'emotion'/exp OR 'fatigue'/exp OR 'energy'/exp OR 'satisfaction'/exp OR 'happiness'/exp OR 'vitality'/exp

PsycINFO

COPD OR "Chronic obstructive pulmonary disease" OR "lung cancer" OR “Lung Neoplasm*” OR “lung tumor*”OR Asthma OR “Chronic bronchitis” OR Emphysema OR “cystic fibrosis” OR ILF OR “idiopathic lung fibrosis” OR “Idiopathic pulmonary fibrosis” OR “fibrosing alveolitis” OR “pulmonary fibrosis” OR “lung fibrosis” OR "Lung injuries” OR "Silicosis” OR “Sleep Apnea” OR “Sleep Apnoea” OR “Pulmonary hypertension” OR Sarcoidosis OR “Pulmonary embolism” OR “lung embolism” OR “Pulmonary arterial hypertension” OR PAH OR “Primary ciliary dyskinesia” OR PCD OR Pneumothorax OR “occupational lung diseases” OR Lymphangioleiomyomatosis OR “Interstitial lung disease” OR “Hypersensitivity pneumonitis” OR “Eosinophilic granulomatosis with polyangiitis” OR Mesothelioma OR “Vaping Use-Associated Lung Injury” OR “E-cigarette Use-Associated Lung Injury” OR EVALI OR “Vaping associated pulmonary illness” OR VAPI OR “Cryptogenic organizing pneumonia” OR COP OR “Black lung disease” OR Anthracosis OR “Bronchopulmonary dysplasia” OR “Lung Dysplasia” OR BPD OR “Bronchiolitis obliterans” OR Bronchiolitis OR “Chronic obstructive airways disease” OR Asbestosis OR “Lung injury” OR “Coal workers' pneumoconiosis” OR CWP OR “Primary ciliary dyskinaesia” OR COAD OR Pneumonia OR “Lung Disorder*” OR "Lung Disease*" OR MAINSUBJECT.EXACT.EXPLODE("Chronic Obstructive Pulmonary Disease") OR MAINSUBJECT.EXACT.EXPLODE("Pulmonary Emphysema") OR MAINSUBJECT.EXACT.EXPLODE("Cystic Fibrosis") OR MAINSUBJECT.EXACT.EXPLODE("Lung Disorders") OR MAINSUBJECT.EXACT.EXPLODE("Sleep Apnea") OR MAINSUBJECT.EXACT.EXPLODE("Pneumonia")

AND

Danc* OR MAINSUBJECT.EXACT("Dance")

AND

Wellbeing OR well-being OR “mental health” OR “physical health” OR health OR “quality of life” OR “QoF scale” OR “Breathing efficiency” OR Balance OR Gait OR anxiety OR fitness OR depression OR “exercise capacity” OR “muscle tone” OR “shortness of breath” OR Dyspnea OR dyspnoea OR emotion* OR tiredness OR Fatigue OR Energy OR Worry OR satisfaction OR happiness OR vitality OR MAINSUBJECT.EXACT.EXPLODE("Well Being") OR MAINSUBJECT.EXACT("Mental Health") OR MAINSUBJECT.EXACT("Physical Health") OR MAINSUBJECT.EXACT("Quality of Life") OR MAINSUBJECT.EXACT("Gait") OR MAINSUBJECT.EXACT("Anxiety Disorders") OR MAINSUBJECT.EXACT("Depression (Emotion)") OR MAINSUBJECT.EXACT("Muscle Tone") OR MAINSUBJECT.EXACT("Dyspnea") OR MAINSUBJECT.EXACT("Emotions") OR MAINSUBJECT.EXACT("Fatigue") OR MAINSUBJECT.EXACT("Satisfaction") OR MAINSUBJECT.EXACT("Satisfaction") OR MAINSUBJECT.EXACT("Dance")
